# Incidence of impacted mandibular and maxillary third molars: a radiographic study in a Southeast Iran population

**DOI:** 10.4317/medoral.18028

**Published:** 2012-12-10

**Authors:** Maryam A. Hashemipour, Mehrnaz Tahmasbi-Arashlow, Farnaz Fahimi-Hanzaei

**Affiliations:** 1 DDS, MSc. Member of Kerman Dental and Oral Diseases Research Center. Department of Oral Medicine, School of Dentistry, Kerman University of Medical Sciences, Kerman, Iran; 2 DDS. Member of Kerman dental and oral diseases research center. Kerman University of Medical Sciences, Kerman, Iran; 3 DDS, MSc. Department of Radiology, University of Medical Sciences, Kerman, Iran

## Abstract

Objectives: The aim of this study is to evaluate the position of impacted third molars based on the classifications of Pell & Gregory and Winter in a sample of Iranian patients. Study design: In this retrospective study, up to 1020 orthopantomograms (OPG) of the patients who were referred to the radiology clinics from October 2007 to January 2011 were evaluated. Data including the age, gender, the angulation type, width and depth of impaction were evaluated by statistical tests. Results: Among 1020 patients, 380(27.3%) were male and 640(62.7%) were female with the sex ratio was 1:1.7. Of the 1020 OPGs, 585 cases showed at least one impacted third molar, with significant difference between males (205; 35.1%) and females (380; 64.9%) (P = 0.0311). Data analysis showed that impacted third molars were 1.9 times more likely to occur in the mandible than in the maxilla (P =0.000). The most common angulation of impaction in the mandible was mesioangular impaction (48.3%) and the most common angulation of impaction in the maxilla was the vertical (45.3%). Impaction in the level IIA was the most common in both maxilla and mandible. There was no significant diffe-rence between the right and left sides in both the maxilla and the mandible. Conclusion: The pattern of third molar impaction in the southeast region of Iran is characterized by a high prevalence of impaction, especially in the mandible. Female more than male have teeth impaction. The most common angulation was the mesioangular in the mandible, and the vertical angulation in the maxilla. The most common level of impaction was the A and there was no any significant difference between the right and left sides in both jaws.

** Key words:**Third molar, impaction, incidence, Iran.

## Introduction

Tooth impaction is a pathological situation in which a tooth can not or will not erupt into its normal functioning position. This problem can be solved by dental treatment (1). The mandibular third molars are the most frequently impacted teeth that can be found in human (2).

The prevalence of third molar impaction ranges from 16.7% to 68.6% (3-10). Most studies have reported no sexual predilection in third molar impaction (3,4,6,8). Some studies, however, have reported a higher frequency in females than males (10,11).

Impacted teeth are often associated with pericoronitis, periodontitis, cystic lesions, neoplasm,root resorption and can cause detrimental effects on adjacent tooth (12).

Also, many research works have shown that impacted third molar weakens the angle of mandible and makes it susceptible to fracture (13,14) and is implicated in the etiology of lower arch crowding, Temporomandibular Joint (TMJ) disorders, vague oro-facial pain and neuralgias (15,16).

Several methods have been used to classify the impaction. This classification is based on many factors which are the level of impaction (15), the angulations of the third molars and the relationship to the anterior border of the ramus of the mandible. Depth or level of maxillary and mandibular third molars can be classified using the Pell and Gregory classification system, where the impacted teeth are assessed according to their relationship to the occlusal surface (OS) of the adjacent second molar (17).

According to the fact that the pattern of third molar impaction has not been assessed in the southeast area of Iran, the objective of this study is to evaluate the pattern of third molar impaction using panoramic radiograph in a sample of patients living in the southeast region of Iran. The null hypothesis tested was that there were no differences between the pattern of third molar impactions and its factors (age, gender, the angulation type, width and depth of impaction) in the southeast of Iran with those reported in several other investigations in different locations through the world.

## Material and Methods

-Study Design

This study was undertaken on 2300 patients, at radiology clinics from October 2007 to January 2011. One thousand twenty orthopantomograms of patients aged 19 years along with their related data were selected from these records. With the consent of the patients, all necessary information about the variables of the study written in a Performa were obtained through historical, clinical examination and radiographic study. The age, sex, number of impacted third molar were recorded.

The position of the impacted third molar was determined by OPG. Records of patients aged younger than 19 years, with any pathological dento-alveolar condition, any craniofacial anomaly or syndrome such as Down syndrome, cleidocranial dysostosis, or with the presence of incomplete records or poor quality OPG, incomplete root formation of third molar, or absence of adjacent second molar, were excluded. OPGs were reviewed by a single examiner in a dark room using an appropriate X-ray viewer to determine the prevalence of impacted third molars in the sample, their levels of eruption; and their angulations. Third molar status was determined based on the patient’s chart and OPG. Third molar was considered impacted if it did not have functional occlusion and at the same time, its roots were fully formed.

**Figure 1:**
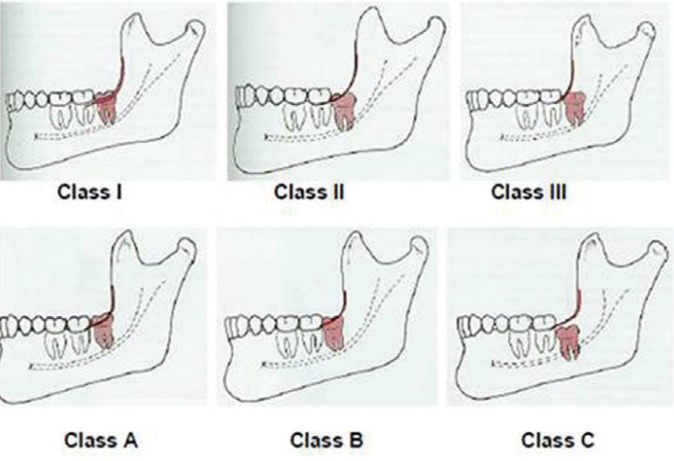
Pell and Gregory classification.

The depth of impacted third molar in relation to occlusal plane [Class A: not buried in bone, or the occlusal plane of the impacted tooth is at the same level as the adjacent tooth, B: partially buried in bone, or the occlusal plane of the impacted tooth is between the occlusal plane and the cervical line of the adjacent tooth (if any part of the cemento-enamel junction was lower than the bone level), C: completely buried in bone, or the occlusal plane of the impacted tooth is apical to the cervical line of the adjacent tooth] was recorded along with the distance or width between the vertical ascending mandibular ramus and the distal surface of the second molar (Class I: situated anterior to the anterior border of the ramus; II: crown half covered by the anterior border of the ramus; III: crown fully covered by the anterior border of the ramus) according to the classification of Pell and Gregory (Fig. 1) (2,17).

**Figure 2:**
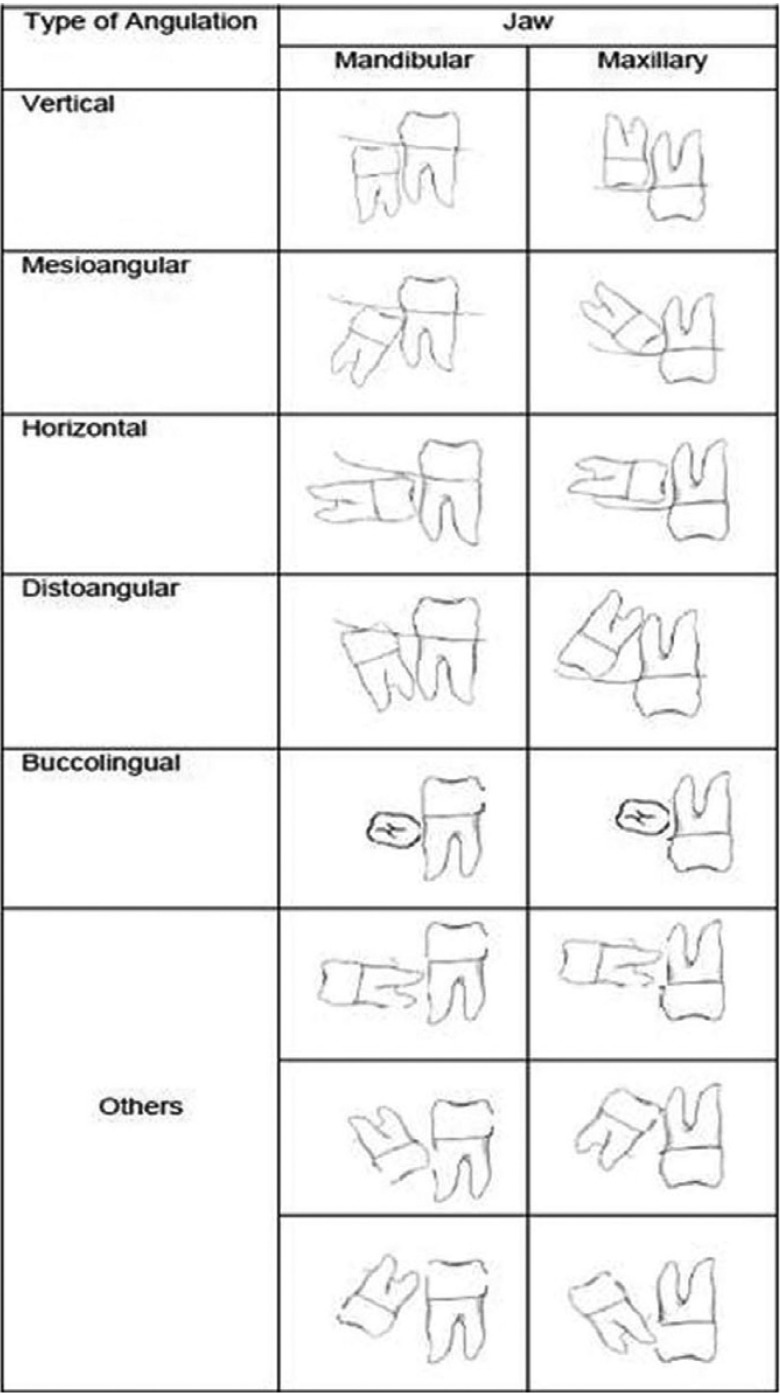
Winter’s classification.

The angulation of impacted third molar was documented based on Winter’s classification with reference to the angle formed between the intersected longitudinal axes of the second and third molars [The vertical impaction (10° to -10°), mesioangular impaction (11° to 79°), horizontal impaction (80° to 100°), distoangular impaction ( -11° to -79°), others (111° to -80°) and buccolingual impaction (Any tooth oriented in a buccolingual direction with crown overlapping the roots)] (Fig. 2) (18).

-Statistical analysis

Data were analyzed using a Pearson chi-square test, performed using the Statistical Package for the Social Sciences (version 18.0; SPSS, Inc, Chicago, IL). The age, gender, number of impacted third molars and classification of impaction were displayed by frequency and percentage. The relations between the groups were analyzed by using the Pearson Chi-square test. The level of significance was 5% (p < 0.05) and data were presented with 95% confidence intervals where applicable. All assessment was done by a single examiner to eliminate the inter-examiner errors. To measure the intra-examiner error, one hundred fifty OPGs were reassessed twice, with a two-week interval, such that its value was calculated equal to 8.2%. All data regarding patient identifica-tion and medical conditions were kept confidential.

## Results

Among 1020 patients, 380 (27.3%) were male and 640 (62.7%) were female; with the sex ratio of 1:1.7. The age range was from 19 to 55 years (mean age ± SD= 26.2 ± 5.8). In the research work, there are five age groups including 11-20, 21-30, 31-40, 41-50 and greater than 50 with the number of patients equal to 30 (2.9%), 568 (55.7%), 225 (22.1%), 150 (13.7%) and 47 (4.6%) respectively.

From the 1020 OPGs, 585 showed at least one impacted third molar, with significant difference between males (205; 35.1%) and females (380; 64.9%) (P = 0.0311). Also, the number of patients with two, three and four impacted third molars were equal to 95 (11.9%), 70 (8.8%), 45 (5.7%). The proportion of impacted mandibular third molars was significantly more than that of impacted maxillary third molars, and more than that of impacted upper and lower third molars together (P =0.000) ( Table 1). Impacted third molars were 1.9 times more likely to occur in the mandible than in the maxilla.

**Table 1:**
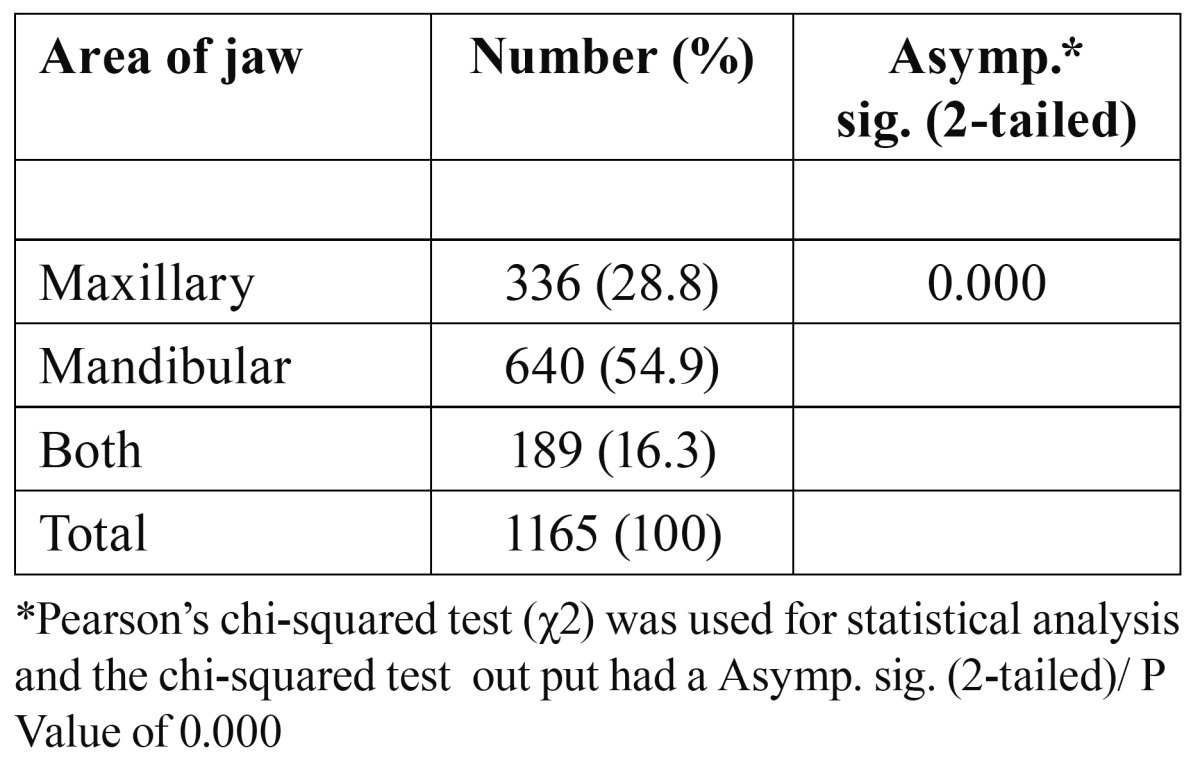
Distribution of impacted teeth by area of the jaw.

The most common angulation of impaction in the mandible was mesioangular impaction (48.3%) followed by horizontal (29.3%), vertical (15.5%) and distoangular impaction (6.3%).The most common angulation of impaction in the maxilla was the vertical (45.3%), followed by the distal(22.2%) ( Table 2).

**Table 2:**
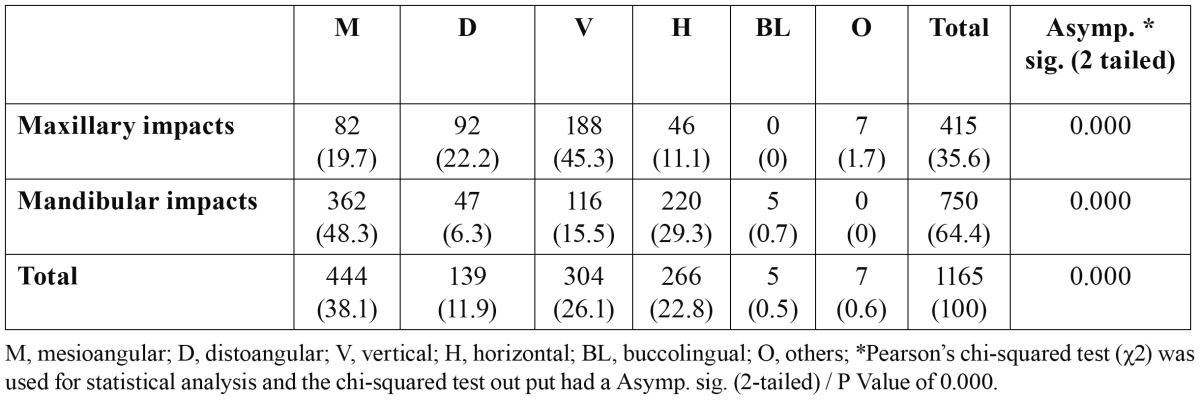
Distribution (%) of third molar impaction by angulations.

Majority of the patients presented with Class IIA (n=510, 43.8%) and IIIC was the least common (n= 29, 2.5%) ( Table 3). There was no significant difference between the right and left sides in both the maxilla (P=0.621) and the mandible (P=0.321) ( Table 4).

**Table 3:**
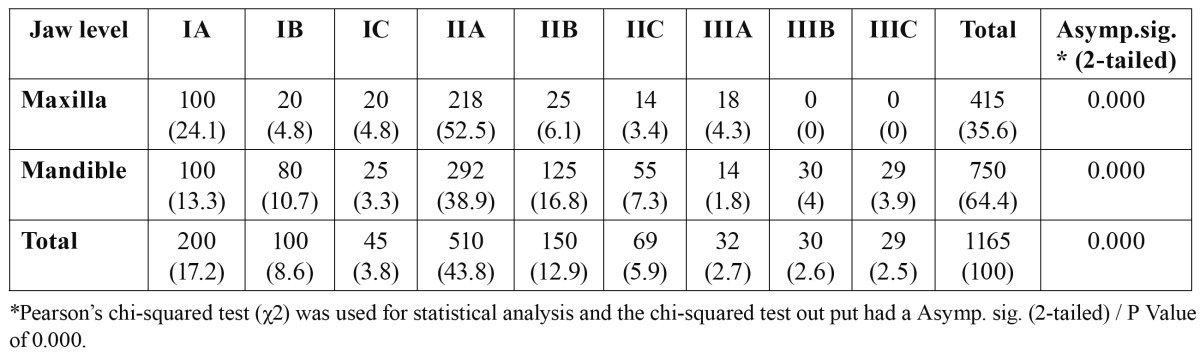
Distribution (%) of third molar impaction by level of impaction.

**Table 4:**
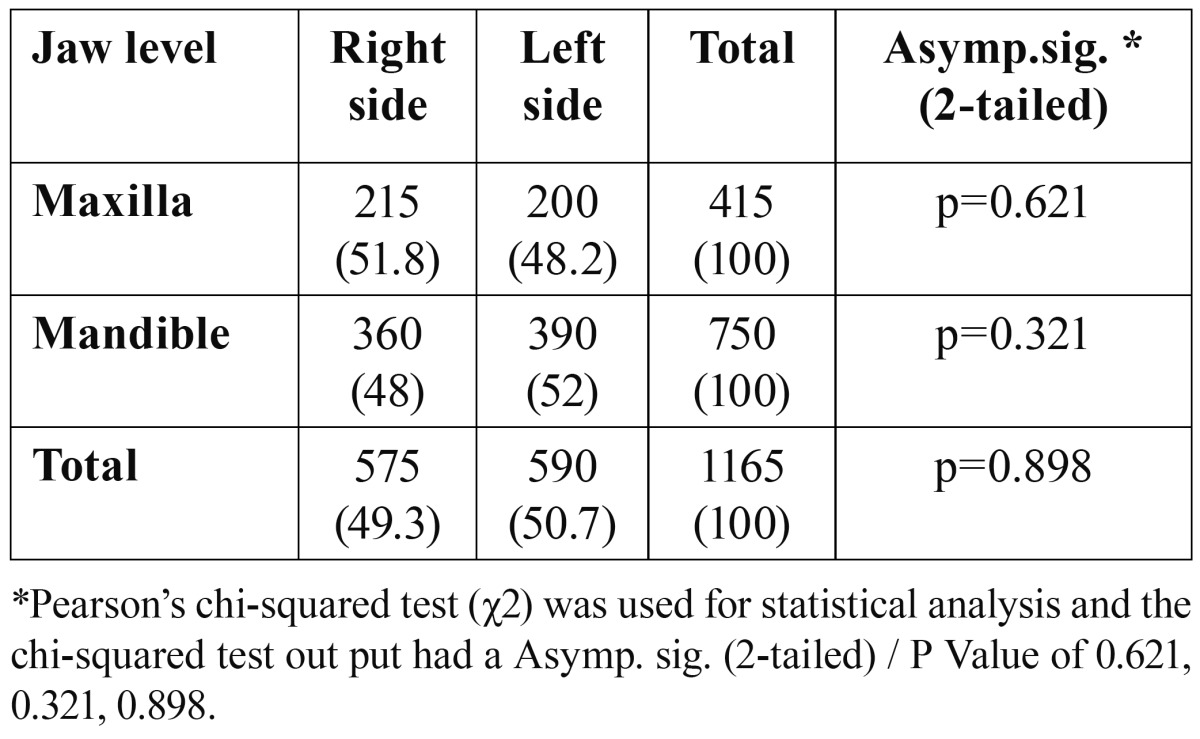
Distribution (%) of third molar impaction by side of impaction.

## Discussion

This study showed that the pattern of third molar impaction in the present sample is characterized by a high prevalence in female. Mesioangular and horizontal type of impaction were the most common. Impaction depth classification of IIA and IA are the teeth most inclined to develop complications.

Third molar impaction is a common problem affecting a large proportion of population through the world. In this study, the incidence of impacted third molars in the Southeast region of Iran was estimated at 44.3% (1020/2300). The reported incidence in the present research work is higher than that reported before by Eliasson et al. (30.3%) (19), Hattab et al. (33%) (4), and Rajasuo et al. (38%) (20). On the other hand, it is less than that reported by Morris and Jerman (21) and Quek et al. (10), who reported frequencies of 65.6% in a study of 5000 subjects in United States of America and 68.6% in a sample of 1000 subjects in Singapore, respectively (1). This difference may be due to the fact that the genetic and racial differences are two important factors in tooth impaction.

In this study, more than half of the patients were in the third decade of their lives, which correlates with the previous studies (12,17). In contrast to the previous investigations, higher proportions of patients (19.3%) were older than 40 years. This may be due to the fact that lack of oral health awareness leads to unnecessary delay in treatment in these patients. This study also revealed that the impaction of third molar was common in female, and this finding was in agreement to previous reports about the gender distribution (10-12,22). The higher frequency reported in females is due to the consequence of difference between the growth of males and females. Females usually stop growing when the third molars just begin to erupt, whereas in males, the growth of the jaws continues during the time of eruption of the third molars, creating more space for third molar eruption (1).

Although, sexual predilection in third molar impaction has not been reported in many investigations (1,3,4,6,8).

In the present work, the common type in the mandibular mesioangular impaction (48.3%) was followed by horizontal (29.3%). It appears that mesioangular impactions are probably the commonest type and this may be due to their late development and maturation, path of eruption and lack of space in mandible at later age.

Our findings conformed to the previous reports from Pakistan, USA, Nigeria, China, Thailand, Spain and Malaysia, where the most common type was mesioangular impactions (1,10,14,23-25). However, some studies show that vertical impaction is the most common (26,27). This could be due to the fact that a different method of classification of angulation was used in these studies.

In the present study, the most common angulation registered in the maxilla was the vertical angulation and this is in agreement with Quek et al. (10). However, it disagrees with Kruger et al. (28) who found that mesioangular impaction was the most fre-quently observed pattern of impaction in the maxilla.

Level of impaction was assessed according to the level of cemento-enamel junction of the third molar relative to the alveolar bone height and not according to the relationship to the occlusal surface of the adjacent second molar. This is more objective since it excludes any normally erupting third molars. The common type of impaction regarding the Pell and Gregory classification was IIA (n=510, 43.3%) followed by IA (n=200, 17.2%). These findings are in accordance with the results of previous studies (1,15). Obiechina et al. (29) reported the common Class as IIA (31%), while Monaco et al. (30) identified the most common position as Class A (56.2%) and Class II (63%) in Italian population. While Blondeau et al. (20) from Canada and Almendros-Marques et al. (27) from Spain reported Class IIB as the most common position of mandibular third molar. Consequently, the findings of the present study are in agreement with a large number of reports that show most impacted third molars were at Class II position, where half of the crown was in the ramus and the position of the highest portion of third molar was at occlusal level, which is Class A.

The etiology of the third molar impaction has been investigated in many international studies. Several factors were reported as possible causes for third molar impaction, including lack of space distal to the permanent second molar, delayed third molar mineralization and early physical maturation (1). Unfortunately, the etiology of third molar impaction has never been investigated in Iran population. Thereby, future studies are required to evaluate the etiology behind this relatively high frequency of third molar impaction especially in the Southeast region of Iran. The present study, like most of the similar previous works about third molar impaction, used a hospital based sample, which lacks randomization. More precise studies are necessary to evaluate the impaction of third molars in a randomized sample representative of Iran. The present study has several limitations such as difficulty in tracing all the dental records notes and OPG. There were also incomplete data in some dental records. Also, more studies are required to evaluate the pattern of third molars in other regions of Iran.
